# Ocean acidification: Global perspectives and India’s path forward

**DOI:** 10.1016/j.isci.2025.113852

**Published:** 2025-10-24

**Authors:** Vysakh S, Sabarinath S, Shijin Ameri, Puneeta Pandey, Akshay Satish, Vijai Dharmamony, Maneesha Vinodini Ramesh

**Affiliations:** 1 Amrita School for Sustainable Futures, Amrita Vishwa Vidyapeetham, Amritapuri, India; 2 Environmental Defense Fund, New York, NY, USA

**Keywords:** Environmental science, Oceanography, Social sciences

## Abstract

Ocean acidification (OA) poses a significant global threat to marine ecosystems, fisheries, and coastal livelihoods. While several countries have established robust monitoring and mitigation strategies, many regions, including India, are still developing comprehensive responses. Given India’s heavy reliance on ocean-based resources, it is crucial to integrate OA considerations into national marine policies to safeguard biodiversity, support sustainable seafood production, and protect vulnerable coastal communities. In alignment with Sustainable Development Goal (SDG) Target 14.3, which calls for enhanced scientific cooperation and monitoring to address OA, this review highlights key gaps in India’s current OA research and policy landscape. It proposes a strategic framework encompassing improved monitoring systems, socio-ecological impact assessments, and targeted policy interventions. By fostering a holistic and collaborative approach, the study aims to strengthen India’s OA resilience and contribute to broader global mitigation efforts.

## Graphical abstract

**Figure:**
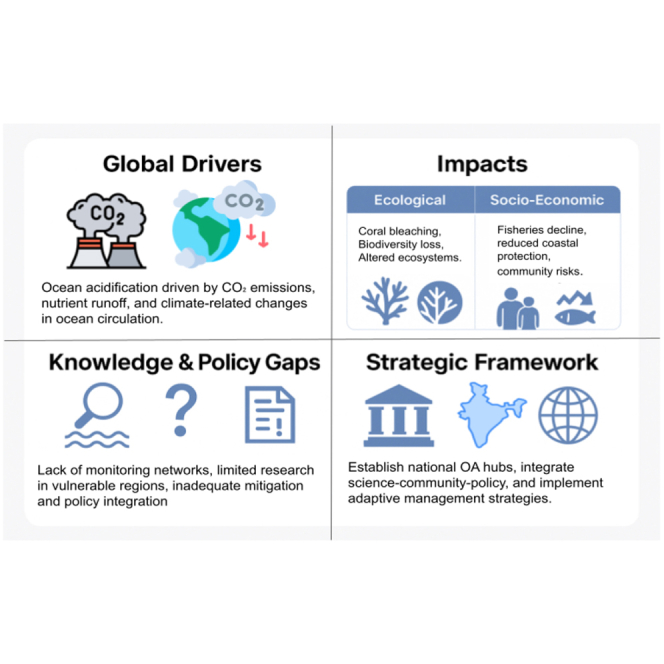

## Introduction

Ocean acidification (OA) is rapidly emerging as one of the most pressing environmental challenges of the 21st century due to its wide-ranging impacts on marine ecosystems, global biodiversity, and human well-being.^1^^,^^2^^,^^3^ Primarily driven by increasing atmospheric carbon dioxide (CO_2_) emissions, OA leads to a reduction in ocean pH and carbonate ion availability, key components for the survival of calcifying organisms such as corals, mollusks, and some planktonic species.^1^^,^^2^^,^^3^ Since the Industrial Revolution, ocean pH has decreased by approximately 0.1 units, making the oceans nearly 30% more acidic, with projections estimating a further drop of 0.3–0.4 by the end of the century if emissions continue unabated.^4^^,^^5^^,^^6^^,^^7^^,^^8^ These changes have significant consequences for marine biodiversity, ecosystem functioning, and the services oceans provide to society, including food security and coastal protection.^9^^,^^10^^,^^11^

The chemical foundation of OA lies in the ocean’s role as a major CO_2_ sink, absorbing nearly 30% of anthropogenic emissions.^3^ When CO_2_ dissolves in seawater, it forms carbonic acid, which dissociates into hydrogen and bicarbonate ions, reducing pH and the availability of carbonate ions necessary for calcification.^3^ In addition to atmospheric CO_2_, other regional stressors such as coastal eutrophication, hypoxia, and freshwater runoff are increasing acidification, especially in nearshore and estuarine environments.^12^^,^^13^ For instance, in regions with heavy nutrient runoff, the decomposition of algal blooms can release CO_2_ and intensify local acidification.^12^ Similarly, monsoon-driven upwelling events and industrial effluents add complexity to OA trends in the Indian Ocean region.^14^^,^^15^

**Figure 1:**
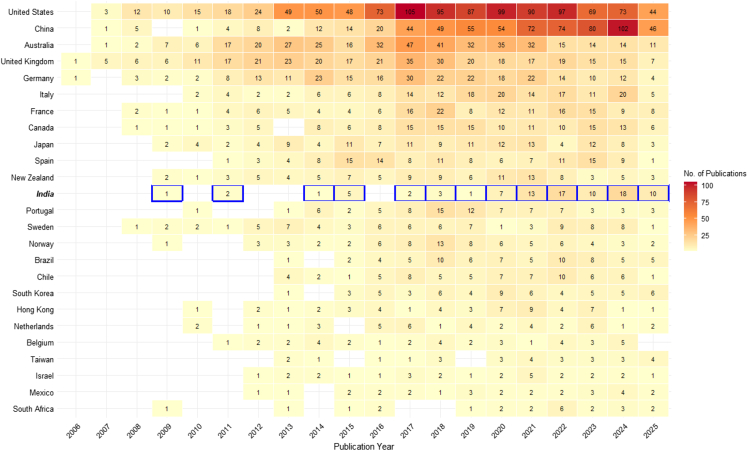
Shade plot of ocean acidification research by country till 2025

In recent years, significant strides have been made globally to understand and respond to OA. Nations such as the United States,^16^ Canada,^17^^,^^18^ Australia,^19^ and Norway,^20^ among others, have developed different large-scale OA monitoring programs. These initiatives include long-term pH and carbonate chemistry observations, breeding of OA-resilient shellfish, artificial reef construction, and marine protected areas (MPAs) to enhance ecosystem resilience.^21^ Potential geoengineering solutions for mitigating the OA impacts include ocean alkalinity enhancement (OAE) and enhanced weathering,^22^^,^^23^ though these have yet to be demonstrated to be large-scale. A bibliometric analysis of global OA research trends, visualized through a shade plot in Figure 1, with the corresponding R code provided in the supplemental files (Code S1), reveals pronounced disparities in research contributions across regions. The United States, China, and Australia consistently dominate OA publications, while India’s research output remains minimal. In recent years, India has seen a slight increase in OA-related studies; however, this growth is marginal compared to global research leaders. The limited number of OA-focused publications from India underscores the absence of a structured OA monitoring network and the lack of dedicated research initiative.

The socio-economic implications of OA in India are particularly significant, especially considering that 67.3% of India’s fishing households live at or below the poverty line.^24^ Limited access to alternative livelihoods and lower adaptive capacity make these communities highly susceptible to disruptions in fisheries and other marine ecosystem services.^25^ Acidification could further contribute to declining trade in commercially valuable species, thus aggravating food security and employment and economic instability problems among fishermen. India has significant coral reef ecosystems in the Gulf of Kachchh, Gulf of Mannar, Lakshadweep, and the Andaman and Nicobar Islands.^26^ While OA poses a serious long-term threat to these reefs, research in India has primarily focused on temperature-driven bleaching, with limited studies on the chemical effects of acidification.

While earlier reviews on OA have established its long-term global significance, recent scientific and policy advances make this an opportune moment for a renewed, regionally grounded assessment. Emerging research since 2020 has applied molecular tools, autonomous observations, and integrated modeling to track organismal and ecosystem responses to acidification more precisely.^2^^,^^10^^,^^17^ In parallel, mitigation actions such as shellfish-selective breeding and blue carbon restoration have gained momentum globally.^20^^,^^27^^,^^28^ For India, a highly vulnerable yet underrepresented region, new studies highlight complex OA drivers such as aerosol deposition, seasonal freshwater variability, and changing monsoon regimes that were poorly understood in earlier literature.^14^^,^^15^^,^^26^ The absence of coordinated national responses, coupled with new ecological data and technological capacity, makes this review both urgent and necessary to guide science-informed action.

Recent studies have provided valuable insights into long-term trends and key drivers of Indian OA over the past four decades using observational and model-based approaches.^29^ Building upon this foundational work, this review synthesizes global and regional knowledge on OA, with a dedicated focus on India’s current scientific understanding, policy landscape, and community vulnerability. We first outline key chemical and physical drivers of OA, followed by illustrative global case studies of ecological and socio-economic impacts. We then provide a detailed assessment of Indian coastal acidification patterns, highlighting regional variability, key threats, and existing research. Finally, we propose a strategic framework for India’s OA resilience, encompassing monitoring infrastructure, research priorities, community engagement, and policy action aligned with Sustainable Development Goal (SDG) 14.3. By situating India’s challenges within a global context, this paper aims to catalyze informed, region-specific responses to one of the most urgent marine issues of our time.

## Methodology

### Design search strategy

This review followed the PRISMA 2020 guidelines to systematically identify, screen, and analyze peer-reviewed literature and key institutional reports on OA. A structured search strategy was developed to ensure comprehensive coverage of relevant studies across global and Indian coastal contexts. The databases Scopus and PubMed were selected for their extensive indexing of scientific literature, complemented by manual searches of institutional repositories. Boolean search strings were constructed using combinations of keywords such as “ocean acidification,” “coastal acidification,” “carbonate chemistry,” “aragonite saturation,” “pCO_2_,” “fisheries,” “impacts,” “mitigation,” and “India.” The final query used for Scopus was TITLE-ABS-KEY (“ocean acidification” OR “coastal acidification”), limited to English-language publications between 2005 and May 2025. This yielded a total of 7,822 records. Following the removal of 3,706 duplicates, the remaining 5,961 studies were imported into the Rayyan platform (https://www.rayyan.ai), a systematic review tool used to streamline blinded screening, tagging, and decision-making. Additionally, six high-quality institutional reports from sources like the Intergovernmental Panel on Climate Change, Global Ocean Acidification Observing Network (GOA-ON) etc. were manually added, given their critical value in providing national and global monitoring perspectives that are often underrepresented in academic databases.

### Inclusion and exclusion criteria

**Figure 2:**
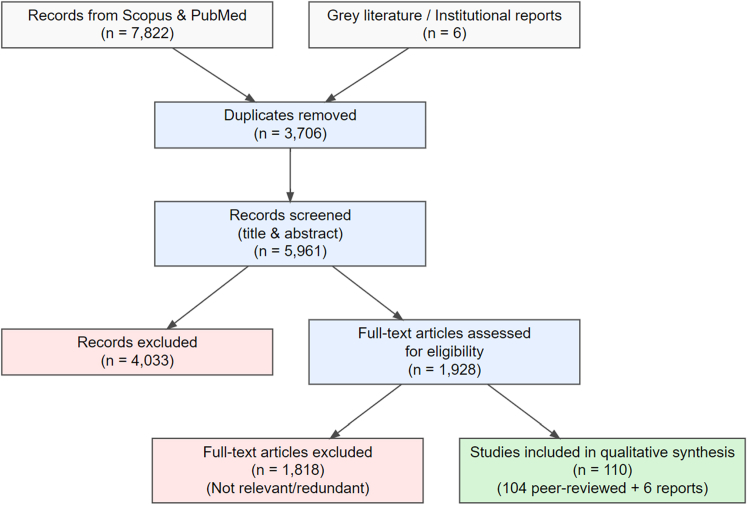
PRISMA flow diagram of study selection for the systematic review, showing identification, screening, eligibility assessment, and final inclusion of 110 studies

To ensure methodological rigor and thematic relevance, a two-stage screening process was implemented. In the initial stage, titles and abstracts of 5,961 articles were reviewed based on predefined inclusion criteria. Eligible studies were required to (1) focus on ocean or coastal acidification in marine or estuarine environments, (2) present empirical measurements or modeling of OA-relevant parameters such as pH, pCO_2_, total alkalinity, dissolved inorganic carbon (DIC), or carbonate saturation states, or (3) assess ecological, biological, or socio-economic impacts related to OA. In the second stage, full-text screening was performed for 1,928 studies identified as potentially relevant. Although a large proportion of these papers were scientifically robust, a final selection of 104 peer-reviewed articles and 6 institutional reports (totaling 110 references) was made. This was done to maintain thematic coherence, reduce redundancy, and align with the review’s comparative and impact-driven scope. The 1,818 studies excluded at this stage were not omitted due to quality concerns but rather because they exhibited thematic overlaps, lacked direct relevance to OA processes, or did not contribute meaningfully to the analytical focus of the review. As shown in Figure 2, this curated selection ensures that the review remains both comprehensive and conceptually focused.

### Review and synthesis method

The selected 110 studies were subjected to qualitative thematic synthesis, categorized under four analytical domains: (1) key drivers and biogeochemical dynamics of OA, (2) ecological and biological responses including calcifying organisms and habitat changes, (3) socio-economic vulnerabilities with a focus on fisheries-dependent livelihoods and adaptive capacities, and (4) mitigation and policy strategies at global and regional levels. Rayyan’s tagging system facilitated the classification of studies based on geographic region, data type (field, experimental, model-based), taxa examined, and OA parameters measured. India-specific studies were given special attention and manually reviewed to extract detailed information on study design, methodology, parameters measured, and findings. Seventeen India-focused papers were identified and compiled into a comparative summary table, enabling spatial and thematic synthesis of OA research within the country. The inclusion of institutional reports offered additional insights into long-term observation networks, national action plans, and early mitigation initiatives. Although no quantitative meta-analysis was performed, the structured mapping approach ensured conceptual clarity and provided a reproducible foundation for identifying knowledge gaps, informing policy discourse, and guiding future research on OA with a particular lens on the Indian subcontinent.

## Key drivers of ocean acidification

**Figure 3:**
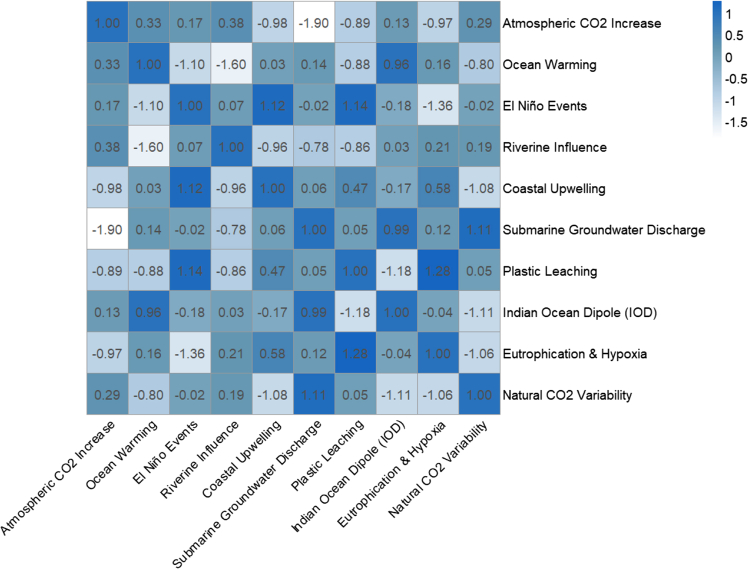
Interaction matrix among ocean acidification drivers

OA is an escalating environmental concern primarily driven by rising concentrations of atmospheric CO_2_ from both anthropogenic and natural sources (Figure 3). The corresponding R code used to generate this visualization is provided in the supplemental files (Code S2), along with the raw dataset (Data S1). The combustion of fossil fuels, deforestation, and industrial processes are the major contributors to rising CO_2_ levels in the atmosphere. Approximately 30% of this CO_2_ is absorbed by ocean waters, where it reacts to form carbonic acid. This weak acid subsequently dissociates into hydrogen (H^+^) and bicarbonate (HCO_3_^−^) ions, decreasing the pH and reducing the availability of carbonate ions (CO_3_^2−^), which are essential for the calcification processes of many marine organisms.^30^^,^^31^^,^^32^ Historical data indicate that industrial carbon emitters, especially fossil fuel producers and the cement industry, are responsible for more than half of the acidification observed at the ocean surface since 1965.^6^^,^^30^^,^^33^ In addition to atmospheric CO_2_, the influx of freshwater from rivers into coastal zones significantly influences ocean acidity. This dilution process alters the carbonate chemistry and buffering capacity of seawater. Major rivers such as the Mississippi-Atchafalaya and Biobío transport large quantities of DIC and nutrients, which modify pH levels and reduce carbonate saturation.^34^^,^^35^ Changes in riverine salinity further disrupt carbonate equilibrium, intensifying acidification in estuarine and coastal waters.^36^^,^^37^^,^^38^

Eutrophication and hypoxia are additional contributors to coastal acidification, particularly in nutrient-rich and semi-enclosed regions such as the northern Gulf of Mexico. Excess nutrients from agriculture and wastewater, mainly nitrogen and phosphorus, stimulate algal blooms. When these algae decompose, microbial respiration releases CO_2_, further lowering pH levels.^12^^,^^39^^,^^40^ This process leads to seasonal hypoxia (low oxygen) and reduced pH, which together threaten marine biodiversity and ecosystem services.

Ocean warming is another critical driver, as it reduces the solubility of CO_2_ in seawater, thereby diminishing the ocean’s capacity to buffer CO_2_ increases.^39^^,^^41^ Additionally, warming alters ocean stratification and circulation, which can influence regional carbonate chemistry, particularly at continental margins.^42^ Submarine groundwater discharge also plays a role in acidification, especially in regions such as the Yucatán Peninsula and Florida, where groundwater rich in CO_2_ is discharged into marine environments.^43^^,^^44^^,^^45^

Seasonal and spatial variations in CO_2_ fluxes, particularly in coastal upwelling zones, also modulate OA intensity. Natural variability in these regions can either exacerbate or mitigate acidification trends^2^^,^^46^; El Niño events significantly influence seawater chemistry by increasing sea surface temperatures, which disrupt carbonate dynamics and cause interannual variability in pH and aragonite saturation.^2^^,^^47^ Similarly, the Indian Ocean Dipole (IOD) intensifies OA in the Indian Ocean by enhancing air-sea CO_2_ exchange and increasing DIC levels.^29^^,^^48^

Notable global upwelling zones, such as those off the coasts of Chile and Australia, transport CO_2_-rich deep waters to the surface, intensifying surface water acidification.^49^^,^^50^ The East Australian Shelf, for instance, has experienced frequent upwelling events, which reduce pH levels and compound stress on marine organisms, particularly coral reefs like the Great Barrier Reef. Reduced carbonate ion concentrations in these areas impede coral calcification and resilience.^51^^,^^52^

A recently emerging contributor to OA is plastic pollution. Studies have shown that the degradation of plastics, especially aged microplastics, can locally reduce seawater pH.^53^ Sunlight accelerates the breakdown of plastics, releasing dissolved organic carbon and organic acids that contribute to acidification.^54^^,^^55^ In heavily polluted coastal zones, accumulated plastics can cause localized acidification effects comparable to long-term global projections.^56^^,^^57^^,^^58^

## Global case studies of ocean acidification impacts

OA has far-reaching ecological and socio-economic consequences across marine ecosystems globally. Its effects span beyond environmental degradation to impact industries like fisheries, aquaculture, tourism, and coastal livelihoods.

**Figure 4:**
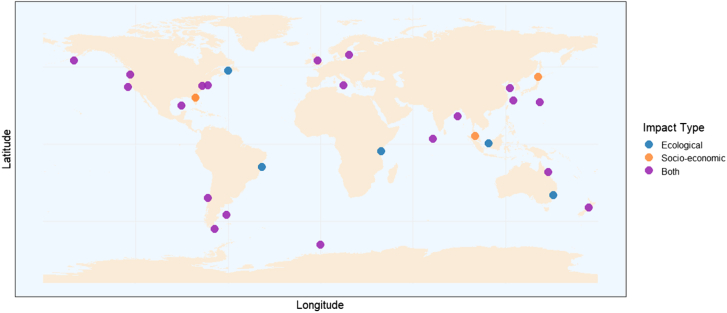
Global distribution of ocean acidification impacts by region and impact type

OA has shown significant ecological and socio-economic impacts across global marine regions as shown in Figure 4. In the Gulf of Mexico,^12^^,^^59^ hypoxia-driven acidification reduces shellfish populations and biodiversity, causing a decline in fisheries that affects local economies and food security, with economic risks for commercial seafood landings, which accounted for $1.4 billion revenue in 2019. The Chesapeake Bay^60^ exhibits reduced biocalcification in oysters, shell dissolution, habitat loss for shellfish, and degradation of oyster reefs, leading to loss of aquaculture revenue and decreased water quality for tourism. The Great Barrier Reef^61^^,^^62^^,^^63^ has experienced coral bleaching and loss of reef-building capacity, with coral calcification rates declining by 11% between 1990 and 2005, the fastest decline in 400 years. This threatens tourism and fisheries industries, with significant economic losses, including AU$162 million annually in fisheries and loss of shoreline protection impacting over 316,000 coastal residents. The Yellow Sea (China)^64^ faces increased shell dissolution, reduced calcification, benthic ecosystem stress, and seasonal hypoxia, creating threats to commercially valuable bivalve and crustacean fisheries and aquaculture. The California Current Large Marine Ecosystem^65^^,^^66^ shows upwelling of CO_2_-rich waters causing extreme pH variations and aragonite undersaturation, leading to biodiversity loss and higher management costs. In the Mediterranean Sea,^67^^,^^68^ a decrease in carbonate concentration affects marine calcifiers such as corals, mollusks, and planktonic organisms, causing structural degradation of habitats like seagrass meadows and coralligenous reefs and disruptions in ecosystem services such as carbon sequestration. This results in declining fisheries yields, aquaculture productivity, negative impacts on red coral extraction for jewelry, and loss of tourism revenue due to reef degradation and harmful algal blooms. Atlantic Canada^69^ reports declines in shellfish populations (lobsters, mussels, oysters, and snow crabs), species distribution shifts, and increased vulnerability to predation and disease, causing economic losses in fisheries, reduced employment, and higher social vulnerability in communities dependent on fisheries. In the US Southeast,^70^ decreased calcification rates in corals, shellfish, and crustaceans, combined with increased toxicity of pesticides and harmful algal blooms, cause major losses in oyster, shrimp, and crab industries, along with reduced ecosystem services such as coastal protection. The US Mid-Atlantic^71^ shows high variability in carbonate chemistry due to estuarine and shelf dynamics, with acidification impacting oysters, finfish, and crustaceans and interactions with hypoxia and eutrophication further intensifying stress, leading to declining fishery productivity and economic risks. In the Pacific Northwest,^21^ high larval mortality in shellfish hatcheries due to elevated CO_2_ levels results in declines in oyster seed production and significant economic losses in oyster farming. The North American West Coast^72^ experiences periodic upwelling of low-pH waters that impact shellfish, finfish, and coral communities, with hypoxia and acidification due to nutrient runoff causing additional stress and economic challenges for fisheries and aquaculture. Similarly, the Northeast Pacific^73^ faces increased vulnerability of salmon and Pacific Halibut due to habitat degradation, harmful algal blooms, and shellfish population declines, with potential negative effects on salmon aquaculture and commercial fisheries. The Gulf Coast and East Coast of the USA^74^ experience significant declines in aragonite saturation states that affect calcifying organisms, leading to increased challenges for aquaculture and fisheries and risks for coastal communities. On the US West Coast,^75^ oyster hatcheries have reported major production failures and reduced larval survival, causing significant economic losses and prompting adaptation strategies such as monitoring, buffering, and shifting production timing. In Asia, the Sunda Shelf Sea (Southeast Asia)^76^ exhibits seasonal acidification driven by extensive remineralization of peatland-derived dissolved organic carbon, reducing seawater pH by 0.10 and causing persistent CO_2_ oversaturation, potentially affecting coral reef health and fisheries. The Bay of Bengal^77^ shows reduced salinity and seasonal acidification, altering marine biodiversity and primary productivity, posing threats to fisheries and the livelihoods of coastal communities. Broader Southeast Asia^40^ reports significant changes in gene expression affecting ion homeostasis and tissue development, posing physiological risks to finfish aquaculture. In the East China Sea,^78^ CO_2_ uptake increases acidification, reducing carbonate availability for calcifying organisms, threatening aquaculture and long-term fisheries productivity. Along the Chilean Coast,^36^^,^^49^ elevated pCO_2_ significantly disrupts filter-feeding behavior in gastropods (*Concholepas concholepas*) and bivalves (*Perumytilus purpuratus*), reducing clearance and ingestion rates by 15%–70%, which could lead to a decline in shellfish fisheries and economic risks for aquaculture. The Brazilian Coast^79^ shows physiological stress in calcified macroalgae species such as *Halimeda cuneata* and *Padina gymnospora*, potentially impacting reef-associated fisheries and marine tourism. In the Beagle Channel,^80^ OA and temperature extremes impair thermal physiology in *Eleginops maclovinus*, leading to oxidative stress, reduced survival, and economic risks for artisanal fisheries. The Patagonian Shelf-Break^81^ experiences decreased aragonite saturation toward undersaturation, impacting marine calcifiers and threatening biodiversity-dependent industries like ecotourism and fisheries. In the Indian Ocean region, Malaysia (Penang & Langkawi)^82^ shows high vulnerability of tropical bryozoans, with 46% of species being calcitic, 41% aragonitic, and 13% bimineralic, creating risks for reef-associated biodiversity and tourism. The East Australian Shelf^51^ faces threats to aragonite-dependent organisms such as corals and mollusks, impacting fisheries and coral reef tourism. The Maldives^44^ reports declining carbonate chemistry and reduced coral calcification, posing risks to reef-associated tourism, fisheries, and coastal protection. The New Zealand Coast^83^ sees reduced calcification and survival in species like green-lipped mussels and pāua (abalone) and losses in reef protection and biodiversity. In Europe, the United Kingdom^84^^,^^85^ is projected to experience a 3%–20% job loss in fisheries and revenue losses of 14%–28% by 2100 due to declining calcification in mollusks and crustaceans. The Baltic-Skagerrak System^86^ faces shifts toward microbial-dominated planktonic systems and reduced calcification in bivalves such as *Mytilus trossulus*, leading to declines in herring and cod stocks, thereby increasing risks to fisheries, aquaculture, and ecosystem services. In Japan, coral reefs around Chichijima and Kikaijima Islands^87^ show rapid declines in coral calcification fluid pH and reduced aragonite saturation, making reefs weaker and more susceptible to bleaching and bioerosion, impacting tourism and fisheries. The Subarctic Coast of Hokkaido^88^ experiences seasonal changes that reduce aragonite saturation, weakening mollusk shells and reducing aquaculture productivity, creating economic risks for fisheries industries. In Kenya (Gazi Bay),^89^ acidification impacts seagrass meadows and mangrove ecosystems, reducing fish populations and increasing coastal vulnerability, with economic losses in tourism and fisheries. Finally, the Southern Ocean^90^ experiences shoaling of the aragonite saturation horizon and reduced carbonate ion concentrations, affecting calcifying organisms such as coccolithophores, foraminifera, and pteropods, with cascading impacts on food webs and krill fisheries. Additionally, reduced carbon uptake may affect the Southern Ocean’s ability to regulate global climate.

## Case studies and drivers of ocean acidification in Indian coastal waters

India’s coastal waters are increasingly vulnerable to OA, driven by a combination of global CO_2_ emissions, monsoonal patterns, regional upwelling, riverine input, and anthropogenic stressors. The Arabian Sea and Bay of Bengal each present unique acidification dynamics shaped by distinct environmental and climatic processes. Long-term model simulations have revealed a significant decline in surface pH (∼0.015 units per decade) across the Arabian Sea and equatorial Indian Ocean, primarily attributed to rising atmospheric CO_2_ concentrations.^29^ In these regions, decreasing total alkalinity and warming sea surface temperatures further intensify acidification. In contrast, the Bay of Bengal shows some buffering effects from riverine alkalinity, although this is counteracted by stratification and warming-induced changes.^29^ Seasonal analyses further show that DIC, temperature, and CO_2_ fluxes drive variations in pH across different bio-provinces, underscoring the spatial heterogeneity of OA.^91^ In the Bay of Bengal, extensive freshwater discharge from major rivers like the Ganges and Godavari leads to strong stratification, which suppresses vertical mixing and traps CO_2_-enriched surface waters. This results in declining pH levels (∼0.003 units per year) and rising pCO_2_, particularly in the western sector.^77^ IOD events further modify coastal acidification by influencing precipitation and runoff patterns. During low discharge years, reduced dilution and enhanced upwelling increase coastal pCO_2_ concentrations.^92^ Experimental studies have demonstrated that marine organisms are already being affected by acidification. For instance, long-term exposure trials on sea urchins in the eastern Arabian Sea showed altered skeletal composition and survivability under low pH conditions, suggesting partial physiological resilience but also vulnerability to chronic acidification.^93^ Similarly, mesocosm experiments off Kochi revealed that elevated pCO_2_ (∼830 μatm) caused significant shifts in phytoplankton biomass and community structure.^94^ Additional experiments with trace metal enrichment (zinc and copper) under acidified conditions revealed species-specific effects, favoring smaller cells and altering food web dynamics.^95^ Atmospheric deposition represents another major acidification driver. Aerosols containing nitrate and sulfate, originating from biomass burning and fossil fuel combustion, contribute to pH reductions, particularly during the dry season in the Bay of Bengal.^15^ In estuarine and urban-influenced coastal zones, shifts in microalgal diversity and composition have been linked to acidification and nutrient over-enrichment, acting as early ecological indicators of stress.^96^ Despite the increasing recognition of OA impacts, systematic monitoring of pH and carbonate chemistry in Indian waters remains sparse. The Indian Ocean, particularly north of 20°S, is among the most poorly observed regions globally for pH, pCO_2_, and total alkalinity.^96^ This data gap limits the ability to validate models and forecast ecological risks. Furthermore, acidification rarely acts in isolation. Multiple stressors such as deoxygenation, eutrophication, and ocean warming often overlap, complicating the prediction of long-term impacts.^97^

These case studies reveal that India’s coastal regions are subject to diverse and interacting acidification drivers. The cumulative influence of global and regional processes, ranging from atmospheric CO_2_ uptake and climate oscillations to river discharge, pollutant input, and biological feedback, creates complex and dynamic acidification patterns. Addressing these challenges requires integrated monitoring networks, long-term biological studies, and the incorporation of OA research into national marine policy and adaptation planning.

## Mitigation strategies for ocean acidification

**Figure 5:**
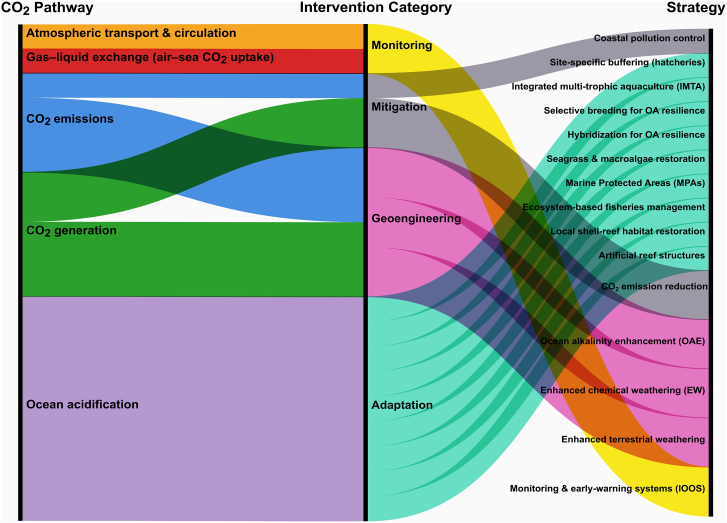
Alluvial diagram showing the linkages between CO_2_ pathways, intervention categories, and specific strategies to address ocean acidification

OA poses disproportionate risks to coastal and island nations, demanding urgent responses. While regions such as the US West Coast have pioneered buffering and selective breeding of OA-resistant shellfish, long-term resilience requires fisheries management, habitat restoration, and global CO_2_ emission reductions. Key strategies are summarized in Figure 5, with comprehensive details presented in Table S1. Global CO_2_ emission reduction remains the cornerstone of mitigation, embedded in international agreements such as the Kyoto Protocol and Paris Agreement.^98^ Strengthening monitoring and early-warning systems^21^^,^^99^ provides the scientific basis for adaptive responses, with networks such as Integrated Ocean Observing System and Pacific Ocean Acidification monitoring enabling detection of OA hotspots and guiding interventions. At regional scales, coastal pollution control^100^ reduces nutrient-driven acidification, linking OA mitigation with broader water-quality management. Emerging geoengineering options include OAE,^22^^,^^23^ enhanced chemical weathering,^101^ and terrestrial weathering.^102^ While these approaches could increase seawater buffering capacity, their scalability and ecological risks remain under investigation. Local adaptation measures offer more immediate relief. These include integrated multi-trophic aquaculture (IMTA),^103^ selective breeding for OA resilience,^104^^,^^105^ seagrass and algae restoration,^106^ and ecosystem-based fisheries management.^107^ Additional strategies such as oyster-reef restoration,^108^ artificial reef structures,^105^ and hybridization for OA tolerance^109^ provide community-scale or experimental pathways to build resilience.

Together, these measures highlight that effective OA mitigation requires combining global emission reductions with regional monitoring, local ecosystem-based interventions, and emerging technological innovations. While CO_2_ emission reduction is the cornerstone, requiring strong political will and international coordination, regional and site-specific approaches such as IMTA, habitat restoration, and selective breeding provide nearer-term benefits but vary in scalability. Embedding these strategies in climate policy and marine spatial planning will maximize their impact. Despite international progress, no coordinated OA mitigation framework currently exists in India.

## Bridging the knowledge gap: The need for ocean acidification research and mitigation strategies in India

The review of mitigation strategies and regional OA impacts highlights a significant knowledge gap in India compared to other major coastal nations. While countries such as the US, Canada, China, and Australia have extensive research on OA’s ecological and socio-economic consequences, India remains underrepresented. Despite having one of the largest fisheries-dependent populations globally, there is limited research on how OA affects key marine resources, including coral reefs, shellfish, and commercial fish species. The Bay of Bengal appears in some studies but primarily in the context of general climate change and primary productivity shifts rather than OA-specific risks.

To address this gap, a region-specific monitoring framework should be developed for India’s diverse coastal and marine ecosystems. Continuous monitoring of OA’s impact on coral reefs and fisheries in the Lakshadweep Islands is crucial, as OA-driven reef degradation reduces reef-building capacity, increases susceptibility to bleaching, and affects fish populations reliant on these habitats. Long-term pH and carbonate chemistry monitoring should be implemented using autonomous sensors. Similarly, the Gulf of Kachchh and the Andaman and Nicobar Islands contain vital coral reef systems that experience multiple stressors, including OA, warming, and pollution. Research should explore how OA interacts with regional environmental factors such as monsoon-driven changes in water chemistry and sedimentation rates. The western and eastern coasts of India also require focused studies. Upwelling zones along the west coast (e.g., Kerala and Karnataka) introduce CO_2_-rich waters, making them natural acidification hotspots. Similarly, riverine discharges along the eastern coast (e.g., Bay of Bengal) bring in high loads of organic matter and nutrients, accelerating acidification. High-frequency observational programs should be established to track seasonal variations in OA drivers and their impact on marine organisms. Estuaries and coastal regions are particularly vulnerable due to river discharge and nutrient runoff from industrial, agricultural, and urban sources, contributing to localized acidification. Long-term field studies should assess the cumulative impacts of these stressors on shellfish populations, fisheries productivity, and aquaculture resilience.

Mitigation efforts should integrate ecological conservation, sustainable fisheries management, and technological interventions. Protecting and cultivating seagrass ecosystems can act as a natural carbon sink, absorbing CO_2_ and helping mitigate OA impacts. Expanding seagrass restoration programs in coastal regions, particularly in Lakshadweep and the Andaman and Nicobar Islands, can enhance marine resilience. Sustainable aquaculture and selective breeding of OA-resilient shellfish species can help sustain fisheries-dependent communities. Strengthening monitoring and early warning systems through continuous pH and carbonate system monitoring using autonomous buoys and satellite-based observation networks is essential for tracking acidification trends and providing timely intervention. Artificial reef structures and coral restoration through reef deployment and transplantation efforts in degraded areas can sustain marine biodiversity and support fisheries.

**Figure 6:**
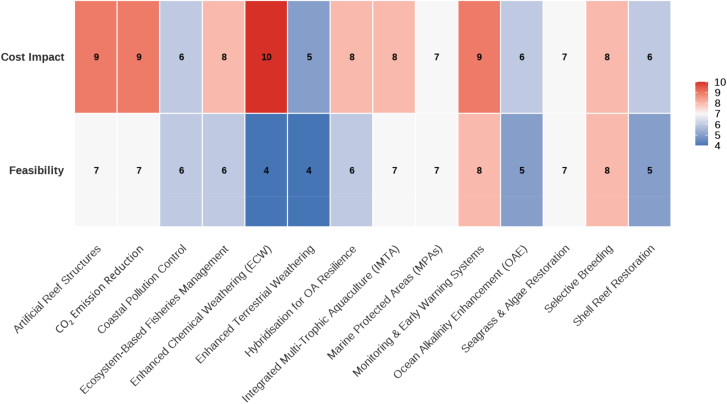
Global ocean acidification mitigation strategies that India can adopt A matrix evaluating global ocean acidification mitigation strategies based on implementation feasibility and effectiveness.

The paper evaluates a range of mitigation strategies, considering both their feasibility and economic implications, as illustrated in Figure 6. The corresponding R code (Code S3) and raw dataset (Data S2) are provided in the supplemental files. Strategies such as CO_2_ emission reduction, monitoring and early warning systems, and MPAs are identified as effective and feasible. Other approaches like OAE and artificial upwelling have higher cost impacts but potential long-term benefits. The feasibility of local habitat restoration, seagrass ecosystem protection, and hybridization for OA resilience is also highlighted, emphasizing the need for region-specific implementation of these strategies.

**Figure 7:**
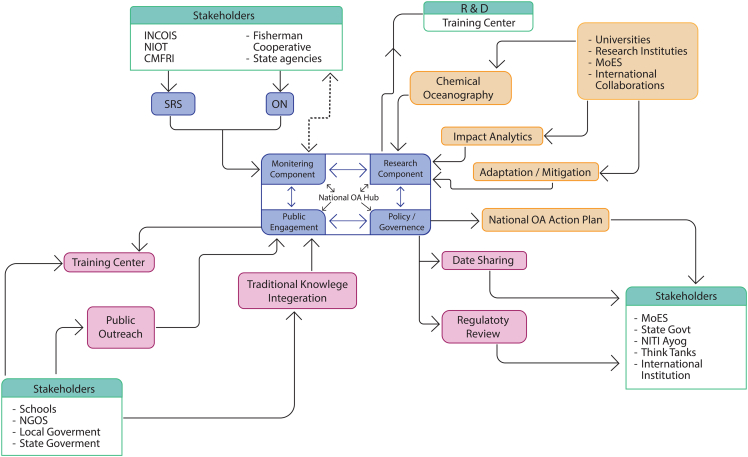
Ocean acidification research and mitigation framework for India A conceptual model outlining a multi-tiered National Ocean Acidification Hub for India.

To implement this strategic approach, establishing a National Ocean Acidification Hub is proposed as conceptualized in Figure 7. The National Ocean Acidification Hub acts as a centralized framework for coordinating India’s scientific, policy, and community responses to the pressing issue of OA. Designed as a multi-tiered structure, the Hub comprises four interconnected components: monitoring, research, public engagement, and policy and governance. These components function in synergy; monitoring generates real-time and long-term data, which feeds into research for deeper analysis and solution development. Research outputs inform the policy and governance component for effective decision-making, while public engagement ensures that both data and policy are grounded in local knowledge and community action. The Hub is positioned to integrate national efforts with global platforms like GOA-ON, UNFCCC, and SDG 14.3, while tailoring approaches to India’s diverse coastal contexts through collaboration with academic institutions, government agencies, NGOs, and citizen groups.

The monitoring component serves as the empirical foundation of the Hub. It collects essential data on ocean chemistry (pH, pCO_2_, dissolved oxygen, and temperature), biological indicators (coral health and shellfish population), and coastal conditions using a combination of coastal observatories, remote sensing, and community-based monitoring. These data are not only crucial for establishing baselines and identifying OA hotspots but also directly inform the research component, enabling scientists to model trends and test hypotheses with real-world evidence. Moreover, localized monitoring insights are translated into community-friendly formats under the public engagement component, fostering ocean literacy and empowering citizens. Continuous data flows from the monitoring arm also feed into the policy and governance component, enabling policymakers to base regulations and strategies on the latest environmental trends and projections.

The research component transforms raw data from monitoring into actionable knowledge. It studies chemical processes (e.g., shifts in the carbonate system), ecological impacts on marine life, and socioeconomic vulnerabilities in affected regions. These research activities are designed to interact dynamically with the other Hub components. For instance, monitoring data feed into modeling and forecasting, while research findings guide the expansion and refinement of monitoring protocols. Research also identifies scalable mitigation strategies like seaweed farming and OA-resilient aquaculture, which are then translated into training programs and local interventions via the public engagement component. Furthermore, research outcomes are synthesized into policy briefs and technical guidelines that shape regulatory frameworks and strategic plans under the policy and governance component, ensuring science-based decision-making.

The public engagement component ensures that scientific knowledge and policy frameworks reach the community level in accessible and impactful ways. It uses tools like citizen science, fisheries awareness programs, school curriculum modules, and community-based adaptation plans to create an informed and resilient public. Importantly, this component acts as a feedback loop: local observations and traditional ecological knowledge collected during engagement feedback into the monitoring component to refine data collection and inform research by highlighting new questions and localized phenomena. Additionally, community responses to pilot mitigation efforts (like seaweed farming or oyster reef restoration) offer ground-level insights for policy refinement. The success of public engagement activities ultimately influences the adoption, effectiveness, and sustainability of OA-related policies developed through the policy and governance component. The policy and governance component institutionalizes the knowledge and practices generated by the other three components. Using data from monitoring and insights from research, this component supports the formulation of a National Ocean Acidification Action Plan, while reviewing and updating relevant regulatory frameworks. Policies are made more inclusive and locally relevant by integrating feedback from public engagement, ensuring that governance is not top-down but participatory. It also builds inter-ministerial coordination across Ministry of Environment, Forest and Climate Change, Ministry of Earth Sciences, and Ministry of Fisheries, promoting a cohesive response to OA. The component strengthens India’s commitments to global initiatives like SDG 14.3 and GOA-ON, while building legal and institutional capacity to manage OA through open data platforms and periodic stakeholder consultations. In this way, it ensures that policy development, scientific knowledge, monitoring infrastructure, and community resilience are mutually reinforcing pillars of the national OA strategy.

## Conclusion and future perspectives

OA is no longer a distant environmental concern; it is a present and accelerating threat to marine biodiversity, fisheries, and the millions who depend on coastal ecosystems for food security and livelihoods. This review has synthesized global scientific understanding of the chemical, ecological, and socio-economic dimensions of OA while contextualizing them within the specific biogeophysical and institutional settings of Indian coastal waters. India’s coasts, influenced by monsoonal cycles, riverine discharge, and atmospheric deposition, are emerging as dynamic zones where acidification trends vary both seasonally and regionally.^14^^,^^15^ Despite this complexity, India remains underrepresented in global OA monitoring efforts, lacking a national framework to observe, interpret, and respond to these changes.^110^ Several research needs have emerged through this synthesis. There is an urgent requirement for high-resolution, long-term pH and carbonate chemistry datasets across India’s marine zones, particularly in upwelling areas, coral reef ecosystems, and estuarine interfaces. Biological assessments remain fragmented, with only limited studies examining physiological and metabolic responses of marine organisms to changing pH levels.^4^^,^^77^ Integrating ecological research with carbonate system modeling and socio-economic vulnerability assessments will be crucial to develop targeted and region-specific mitigation plans. India must prioritize the establishment of a dedicated OA monitoring network, integrated with global platforms such as GOA-ON and aligned with the objectives of SDG 14.3.^110^ This network should incorporate autonomous sensors, coastal observatories, and community-based monitoring programs to ensure both scientific rigor and public participation. At the policy level, OA needs to be mainstreamed into national marine spatial planning, fisheries management, and climate adaptation frameworks. Coastal states should also be supported in formulating subnational responses tailored to local ecological and socio-economic conditions. India is well positioned to take a leadership role in the Global South by developing and sharing models for inclusive, low-cost, and scalable OA monitoring and mitigation strategies. Initiatives such as seagrass restoration, OA-resilient aquaculture, selective breeding of shellfish, and public education programs can serve as replicable models. Leveraging its strong academic network, marine biodiversity, and a large community of ocean-dependent populations, India has the opportunity to transform from a data-deficient region into a proactive stakeholder in the global OA governance landscape.

In conclusion, OA presents not only a scientific challenge but also a policy and community imperative. Addressing it requires a transdisciplinary, multi-stakeholder approach that blends research, governance, and societal engagement. The roadmap proposed in this review offers a pathway for India to respond decisively by advancing monitoring infrastructure, supporting vulnerable communities, and aligning national priorities with global sustainability goals.

## Acknowledgments

We would like to express our immense gratitude to our beloved Chancellor, Dr. Sri. Mata Amritanandamayi Devi (AMMA), for being the guiding light and inspiration for carrying out this work. This project has been funded by the E4LIFE International Ph.D. Fellowship Program offered by 10.13039/100009526Amrita Vishwa Vidyapeetham. We extend our gratitude to the Amrita Live-in-Labs academic program for providing all the support. We are also sincerely thankful to the anonymous reviewers for their constructive feedback, which has greatly improved the quality of this work.

## Author contributions

V.S. led the conceptual development, research design, comprehensive literature review, and drafting of the manuscript. S.S. played a major role in shaping the analytical framework, critically reviewing and refining the manuscript, and guiding the overall structure and clarity. S.A. contributed to contextual research and thematic analysis. A.S. supported data visualization and figure development. P.P. assisted in content integration and writing. V.D. and M.V.R. provided strategic oversight, supervision, and project guidance. All authors reviewed and approved the final version of the manuscript.

## Declaration of interests

The authors declare no competing interests.

## Declaration of generative AI and AI-assisted technologies in the writing process

During the preparation of this work, the author(s) used AI tools to refine language, enhance clarity, and improve the overall coherence of the manuscript. After using this tool, the author(s) reviewed and edited the content as needed and take(s) full responsibility for the content of the publication.

## Supplemental information

Data S1. Supplementary research data for the study

## References

[1] Branch T.A., DeJoseph B.M., Ray L.J., Wagner C.A. (2013). Impacts of ocean acidification on marine seafood. Trends Ecol. Evol..

[2] Ma D., Gregor L., Gruber N. (2023). Four Decades of Trends and Drivers of Global Surface Ocean Acidification. Glob. Biogeochem. Cycles.

[3] Feely R.A., Sabine C.L., Lee K., Berelson W., Kleypas J., Fabry V.J., Millero F.J. (2004). Impact of Anthropogenic CO_2_ on the CaCO_3_ System in the Oceans. Science.

[4] Havenhand J.N., Buttler F.-R., Thorndyke M.C., Williamson J.E. (2008). Near-future levels of ocean acidification reduce fertilization success in a sea urchin. Curr. Biol..

[5] Abbasi T., Abbasi S.A. (2011). Ocean Acidification: The Newest Threat to the Global Environment. Crit. Rev. Environ. Sci. Technol..

[6] Feely R., Doney S., Cooley S. (2009). Ocean acidification: Present conditions and future changes in a high-CO_2_ world. Oceanography (Wash. D. C.).

[7] Orr J.C., Fabry V.J., Aumont O., Bopp L., Doney S.C., Feely R.A., Gnanadesikan A., Gruber N., Ishida A., Joos F. (2005). Anthropogenic ocean acidification over the twenty-first century and its impact on calcifying organisms. Nature.

[8] Bindoff N.L., Cheung W.W.L., Kairo J.G., Arístegui J., Guinder V.A., Hallberg R., Hilmi N., Jiao N., Karim M.S., Levin L. (2019).

[9] Leung J.Y.S., Zhang S., Connell S.D. (2022). Is Ocean Acidification Really a Threat to Marine Calcifiers? A Systematic Review and Meta-Analysis of 980+ Studies Spanning Two Decades. Small.

[10] Kapsenberg L., Bitter M.C., Miglioli A., Aparicio-Estalella C., Pelejero C., Gattuso J.-P., Dumollard R. (2022). Molecular basis of ocean acidification sensitivity and adaptation in Mytilus galloprovincialis. iScience.

[11] Gattuso J.-P., Magnan A., Billé R., Cheung W.W.L., Howes E.L., Joos F., Allemand D., Bopp L., Cooley S.R., Eakin C.M. (2015). Contrasting futures for ocean and society from different anthropogenic CO_2_ emissions scenarios. Science.

[12] Cai W.-J., Hu X., Huang W.-J., Murrell M.C., Lehrter J.C., Lohrenz S.E., Chou W.-C., Zhai W., Hollibaugh J.T., Wang Y. (2011). Acidification of subsurface coastal waters enhanced by eutrophication. Nat. Geosci..

[13] Breitburg D., Levin L.A., Oschlies A., Grégoire M., Chavez F.P., Conley D.J., Garçon V., Gilbert D., Gutiérrez D., Isensee K. (2018). Declining oxygen in the global ocean and coastal waters. Science.

[14] Sarma V.V.S.S., Krishna M.S., Paul Y.S., Murty V.S.N. (2015). Observed changes in ocean acidity and carbon dioxide exchange in the coastal Bay of Bengal – a link to air pollution. Tellus B.

[15] Sarma V.V.S.S., Krishna M.S., Srinivas T.N.R., Kumari V.R., Yadav K., Kumar M.D. (2021). Elevated Acidification Rates Due to Deposition of Atmospheric Pollutants in the Coastal Bay of Bengal. Geophys. Res. Lett..

[16] Rosenau N.A., Galavotti H., Yates K.K., Bohlen C.C., Hunt C.W., Liebman M., Brown C.A., Pacella S.R., Largier J.L., Nielsen K.J. (2021). Integrating High-Resolution Coastal Acidification Monitoring Data Across Seven United States Estuaries. Front. Mar. Sci..

[17] Moran K., Juniper S.K., Bligh S., Loock D., Kulin I., Paulson M., Pirenne B. (2022). Canada’s Internet-Connected Ocean. Front. Mar. Sci..

[18] Gibb O., Cyr F., Azetsu-Scott K., Chassé J., Childs D., Gabriel C.-E., Galbraith P.S., Maillet G., Pepin P., Punshon S., Starr M. (2023). Spatiotemporal variability in pH and carbonate parameters on the Canadian Atlantic continental shelf between 2014 and 2022. Earth Syst. Sci. Data.

[19] Fabricius K.E., Neill C., Van Ooijen E., Smith J.N., Tilbrook B. (2020). Progressive seawater acidification on the Great Barrier Reef continental shelf. Sci. Rep..

[20] Skjelvan I., Lauvset S.K., Johannessen T., Gundersen K., Skagseth Ø. (2022). Decadal trends in Ocean Acidification from the Ocean Weather Station M in the Norwegian Sea. J. Mar. Syst..

[21] Barton A., Waldbusser G., Feely R., Weisberg S., Newton J., Hales B., Cudd S., Eudeline B., Langdon C., Jefferds I. (2015). Impacts of Coastal Acidification on the Pacific Northwest Shellfish Industry and Adaptation Strategies Implemented in Response. Oceanography (Wash. D. C.).

[22] Oschlies A., Bach L.T., Rickaby R.E.M., Satterfield T., Webb R., Gattuso J.-P. (2023). Climate targets, carbon dioxide removal, and the potential role of ocean alkalinity enhancement. State Planet.

[23] Butenschön M., Lovato T., Masina S., Caserini S., Grosso M. (2021). Alkalinization Scenarios in the Mediterranean Sea for Efficient Removal of Atmospheric CO_2_ and the Mitigation of Ocean Acidification. Front. Clim..

[24] Central Marine Fisheries Institute-CMFRI (2020). Marine Fisheries Census 2016 - India.

[25] Syda Rao G., Sathianandan T.V., Kuriakose S., Mini K.G., Najmudeen T.M., Jayasankar J., Mathew W.T. (2016). Demographic and socio-economic changes in the coastal fishing community of India. Indian J. Fish..

[26] Thinesh T., De K., Sobanaa M., Sivagurunathan P., Sahayariana P., Ramasamy P., Selvin J., Jose P.A., Bellantuono A.J. (2025). History of recurrent short- and long-term coral bleaching events in Indian coral reefs: A systematic review of contrasting bleaching patterns, lessons learned, and future directions. Estuar. Coast Shelf Sci..

[27] Feng J.-C., Sun L., Yan J. (2023). Carbon sequestration via shellfish farming: A potential negative emissions technology. Renew. Sustain. Energy Rev..

[28] Anthony K.R.N., Kline D.I., Diaz-Pulido G., Dove S., Hoegh-Guldberg O. (2008). Ocean acidification causes bleaching and productivity loss in coral reef builders. Proc. Natl. Acad. Sci. USA.

[29] Chakraborty K., Joshi A.P., Ghoshal P.K., Baduru B., Valsala V., Sarma V.V.S.S., Metzl N., Gehlen M., Chevallier F., Lo Monaco C. (2024). Indian Ocean Acidification and Its Driving Mechanisms Over the Last Four Decades (1980–2019). Glob. Biogeochem. Cycles.

[30] Doney S.C., Fabry V.J., Feely R.A., Kleypas J.A. (2009). Ocean Acidification: The Other CO2 Problem. Ann. Rev. Mar. Sci.

[31] Bates N., Astor Y., Church M., Currie K., Dore J., Gonaález-Dávila M., Lorenzoni L., Muller-Karger F., Olafsson J., Santa-Casiano M. (2014). A Time-Series View of Changing Ocean Chemistry Due to Ocean Uptake of Anthropogenic CO_2_ and Ocean Acidification. Oceanography (Wash. D. C.).

[32] Friedlingstein P., O’Sullivan M., Jones M.W., Andrew R.M., Gregor L., Hauck J., Le Quéré C., Luijkx I.T., Olsen A., Peters G.P. (2022). Global Carbon Budget 2022. Earth Syst. Sci. Data.

[33] Licker R., Ekwurzel B., Doney S.C., Cooley S.R., Lima I.D., Heede R., Frumhoff P.C. (2019). Attributing ocean acidification to major carbon producers. Environ. Res. Lett..

[34] Sawall Y., Harris M., Lebrato M., Wall M., Feng E.Y. (2020). Discrete Pulses of Cooler Deep Water Can Decelerate Coral Bleaching During Thermal Stress: Implications for Artificial Upwelling During Heat Stress Events. Front. Mar. Sci..

[35] Duarte C.M., Hendriks I.E., Moore T.S., Olsen Y.S., Steckbauer A., Ramajo L., Carstensen J., Trotter J.A., McCulloch M. (2013). Is Ocean Acidification an Open-Ocean Syndrome? Understanding Anthropogenic Impacts on Seawater pH. Estuaries Coasts.

[36] Vargas C.A., Aguilera V.M., Martín V.S., Manríquez P.H., Navarro J.M., Duarte C., Torres R., Lardies M.A., Lagos N.A. (2015). CO_2_-Driven Ocean Acidification Disrupts the Filter Feeding Behavior in Chilean Gastropod and Bivalve Species from Different Geographic Localities. Estuaries Coasts.

[37] Feely R.A., Alin S.R., Newton J., Sabine C.L., Warner M., Devol A., Krembs C., Maloy C. (2010). The combined effects of ocean acidification, mixing, and respiration on pH and carbonate saturation in an urbanized estuary. Estuar. Coast Shelf Sci..

[38] Salisbury J., Green M., Hunt C., Campbell J. (2008). Coastal Acidification by Rivers:A Threat to Shellfish?. EoS Transactions.

[39] Waldbusser G.G., Salisbury J.E. (2014). Ocean Acidification in the Coastal Zone from an Organism’s Perspective: Multiple System Parameters, Frequency Domains, and Habitats. Ann. Rev. Mar. Sci.

[40] Wang L., Sun F., Wen Y., Yue G.H. (2021). Effects of Ocean Acidification on Transcriptomes in Asian Seabass Juveniles. Mar. Biotechnol..

[41] Gruber N. (2011). Warming up, turning sour, losing breath: ocean biogeochemistry under global change. Philos. Trans. A Math. Phys. Eng. Sci..

[42] Kwiatkowski L., Torres O., Bopp L., Aumont O., Chamberlain M., Christian J.R., Dunne J.P., Gehlen M., Ilyina T., John J.G. (2020). Twenty-first century ocean warming, acidification, deoxygenation, and upper-ocean nutrient and primary production decline from CMIP6 model projections. Biogeosciences.

[43] Crook E.D., Potts D., Rebolledo-Vieyra M., Hernandez L., Paytan A. (2012). Calcifying coral abundance near low-pH springs: implications for future ocean acidification. Coral Reefs.

[44] Liu Y.-W., Lin K., Morgan K., Wang X. (2024). Ocean acidification in the tropical Indian Ocean over the past 37 years: Insights from δ^11^B and B/Ca records in a Maldives coral. Chem. Geol..

[45] Crook E.D., Cohen A.L., Rebolledo-Vieyra M., Hernandez L., Paytan A. (2013). Reduced calcification and lack of acclimatization by coral colonies growing in areas of persistent natural acidification. Proc. Natl. Acad. Sci. USA.

[46] Landschützer P., Gruber N., Bakker D.C.E. (2016). Decadal variations and trends of the global ocean carbon sink. Glob. Biogeochem. Cycles.

[47] Sutton A.J., Sabine C.L., Feely R.A., Cai W.-J., Cronin M.F., McPhaden M.J., Morell J.M., Newton J.A., Noh J.-H., Ólafsdóttir S.R. (2016). Using present-day observations to detect when anthropogenic change forces surface ocean carbonate chemistry outside preindustrial bounds. Biogeosciences.

[48] Wang G., Cai W., Santoso A., Abram N., Ng B., Yang K., Geng T., Doi T., Du Y., Izumo T. (2024). The Indian Ocean Dipole in a warming world. Nat. Rev. Earth Environ..

[49] Vargas C.A., Contreras P.Y., Pérez C.A., Sobarzo M., Saldías G.S., Salisbury J. (2016). Influences of riverine and upwelling waters on the coastal carbonate system off Central Chile and their ocean acidification implications. JGR. Biogeosciences.

[50] Feely R.A., Sabine C.L., Hernandez-Ayon J.M., Ianson D., Hales B. (2008). Evidence for Upwelling of Corrosive “Acidified” Water onto the Continental Shelf. Science.

[51] Schulz K.G., Hartley S., Eyre B. (2019). Upwelling Amplifies Ocean Acidification on the East Australian Shelf: Implications for Marine Ecosystems. Front. Mar. Sci..

[52] Chan F., Barth J., Kroeker K., Lubchenco J., Menge B. (2019). The dynamics and impact of ocean acidification and hypoxia. Oceanography (Wash. D. C.).

[53] Romera-Castillo C., Lucas A., Mallenco-Fornies R., Briones-Rizo M., Calvo E., Pelejero C. (2023). Abiotic plastic leaching contributes to ocean acidification. Sci. Total Environ..

[54] Andrady A.L. (2011). Microplastics in the marine environment. Mar. Pollut. Bull..

[55] Ward C.P., Armstrong C.J., Walsh A.N., Jackson J.H., Reddy C.M. (2019). Sunlight Converts Polystyrene to Carbon Dioxide and Dissolved Organic Carbon. Environ. Sci. Technol. Lett..

[56] Jambeck J.R., Geyer R., Wilcox C., Siegler T.R., Perryman M., Andrady A., Narayan R., Law K.L. (2015). Plastic waste inputs from land into the ocean. Science.

[57] Vighi M., Bayo J., Fernández-Piñas F., Gago J., Gómez M., Hernández-Borges J., Herrera A., Landaburu J., Muniategui-Lorenzo S., Muñoz A.-R. (2021). Micro and nano-plastics in the environment: Research priorities for the near future. Rev. Environ. Contam. Toxicol..

[58] Gerritse J., Leslie H.A., de Tender C.A., Devriese L.I., Vethaak A.D. (2020). Fragmentation of plastic objects in a laboratory seawater microcosm. Sci. Rep..

[59] Osborne E., Hu X., Hall E.R., Yates K., Vreeland-Dawson J., Shamberger K., Barbero L., Martin Hernandez-Ayon J., Gomez F.A., Hicks T. (2022). Ocean acidification in the Gulf of Mexico: Drivers, impacts, and unknowns. Prog. Oceanogr..

[60] Waldbusser G.G., Voigt E.P., Bergschneider H., Green M.A., Newell R.I.E. (2011). Biocalcification in the Eastern Oyster (Crassostrea virginica) in Relation to Long-term Trends in Chesapeake Bay pH. Estuaries Coasts.

[61] Smith J.N., Mongin M., Thompson A., Jonker M.J., De’ath G., Fabricius K.E. (2020). Shifts in coralline algae, macroalgae, and coral juveniles in the Great Barrier Reef associated with present-day ocean acidification. Glob. Chang. Biol..

[62] Pendleton L., Hoegh-Guldberg O., Albright R., Kaup A., Marshall P., Marshall N., Fletcher S., Haraldsson G., Hansson L. (2019). The Great Barrier Reef: Vulnerabilities and solutions in the face of ocean acidification. Reg. Stud. Mar. Sci..

[63] Mongin M., Baird M.E., Tilbrook B., Matear R.J., Lenton A., Herzfeld M., Wild-Allen K., Skerratt J., Margvelashvili N., Robson B.J. (2016). The exposure of the Great Barrier Reef to ocean acidification. Nat. Commun..

[64] Li C.L., Yang D.Z., Zhai W.D. (2022). Effects of warming, eutrophication and climate variability on acidification of the seasonally stratified North Yellow Sea over the past 40 years. Sci. Total Environ..

[65] Bednaršek N., Feely R.A., Reum J.C.P., Peterson B., Menkel J., Alin S.R., Hales B. (2014). Limacina helicina shell dissolution as an indicator of declining habitat suitability owing to ocean acidification in the California Current Ecosystem. Proc. R. Soc. A B..

[66] Feely R.A., Alin S.R., Carter B., Bednaršek N., Hales B., Chan F., Hill T.M., Gaylord B., Sanford E., Byrne R.H. (2016). Chemical and biological impacts of ocean acidification along the west coast of North America. Estuar. Coast Shelf Sci..

[67] Rodrigues L.C., van den Bergh J.C.J.M., Ghermandi A. (2013). Socio-economic impacts of ocean acidification in the Mediterranean Sea. Mar. Pol..

[68] Gazeau F., Alliouane S., Bock C., Bramanti L., López Correa M., Gentile M., Hirse T., Pörtner H.-O., Ziveri P. (2014). Impact of ocean acidification and warming on the Mediterranean mussel (Mytilus galloprovincialis). Front. Mar. Sci..

[69] Wilson T.J.B., Cooley S.R., Tai T.C., Cheung W.W.L., Tyedmers P.H. (2020). Potential socioeconomic impacts from ocean acidification and climate change effects on Atlantic Canadian fisheries. PLoS One.

[70] Hall E.R., Wickes L., Burnett L.E., Scott G.I., Hernandez D., Yates K.K., Barbero L., Reimer J.J., Baalousha M., Mintz J. (2020). Acidification in the US Southeast: Causes, potential consequences and the role of the southeast ocean and coastal acidification network. Front. Mar. Sci..

[71] Saba G.K., Goldsmith K.A., Cooley S.R., Grosse D., Meseck S.L., Miller A.W., Phelan B., Poach M., Rheault R., St Laurent K. (2019). Recommended priorities for research on ecological impacts of ocean and coastal acidification in the US Mid-Atlantic. Estuar. Coast Shelf Sci..

[72] Boehm A., Jacobson M., O’Donnell M.J., Sutula M., Wakefield W.W., Weisberg S., Whiteman E. (2015). Ocean acidification science needs for natural resource managers of the North American west coast. Oceanography (Wash. D. C.).

[73] Haigh R., Ianson D., Holt C.A., Neate H.E., Edwards A.M. (2015). Effects of ocean acidification on temperate coastal marine ecosystems and fisheries in the Northeast Pacific. PLoS One.

[74] Wanninkhof R., Barbero L., Byrne R., Cai W.-J., Huang W.-J., Zhang J.-Z., Baringer M., Langdon C. (2015). Ocean acidification along the Gulf Coast and East Coast of the USA. Cont. Shelf Res..

[75] Mabardy R.A., Waldbusser G.G., Conway F., Olsen C.S. (2015). Perception and response of the US west coast shellfish industry to ocean acidification: the voice of the canaries in the coal mine. J. Shellfish Res..

[76] Zhou Y., Evans C.D., Chen Y., Chang K.Y.W., Martin P. (2021). Extensive remineralization of peatland-derived dissolved organic carbon and ocean acidification in the Sunda Shelf Sea, Southeast Asia. JGR. Oceans.

[77] Sridevi B., Sarma V. (2021). Role of river discharge and warming on ocean acidification and pCO_2_ levels in the Bay of Bengal. Tellus B.

[78] Zhang H., Wang K. (2019). Simulated CO_2_-induced Ocean acidification for ocean in the East China: historical conditions since preindustrial time and future scenarios. Sci. Rep..

[79] Scherner F., Pereira C.M., Duarte G., Horta P.A., Castro C.B., Barufi J.B., Pereira S.M.B. (2016). Effects of ocean acidification and temperature increases on the photosynthesis of tropical reef calcified macroalgae. PLoS One.

[80] Lattuca M.E., Vanella F.A., Malanga G., Rubel M.D., Manríquez P.H., Torres R., Alter K., Marras S., Peck M.A., Domenici P., Fernández D.A. (2023). Ocean acidification and seasonal temperature extremes combine to impair the thermal physiology of a sub-Antarctic fish. Sci. Total Environ..

[81] Orselli I.B.M., Kerr R., Ito R.G., Tavano V.M., Mendes C.R.B., Garcia C.A.E. (2018). How fast is Patagonian shelf-break acidifying?. J. Mar. Syst..

[82] Taylor P.D., Tan Shau-Hwai A., Kudryavstev A.B., Schopf J.W. (2016). Carbonate mineralogy of a tropical bryozoan biota and its vulnerability to ocean acidification. Mar. Biol. Res..

[83] Law C.S., Bell J.J., Bostock H.C., Cornwall C.E., Cummings V.J., Currie K., Davy S.K., Gammon M., Hepburn C.D., Hurd C.L. (2018). Ocean acidification in New Zealand waters: trends and impacts. N. Z. J. Mar. Freshw. Res..

[84] Fernandes J.A., Papathanasopoulou E., Hattam C., Queirós A.M., Cheung W.W.W.L., Yool A., Artioli Y., Pope E.C., Flynn K.J., Merino G. (2017). Estimating the ecological, economic and social impacts of ocean acidification and warming on UK fisheries. Fish Fish..

[85] Mangi S.C., Lee J., Pinnegar J.K., Law R.J., Tyllianakis E., Birchenough S.N.R. (2018). The economic impacts of ocean acidification on shellfish fisheries and aquaculture in the United Kingdom. Environ. Sci. Pol..

[86] Havenhand J.N., Filipsson H.L., Niiranen S., Troell M., Crépin A.-S., Jagers S., Langlet D., Matti S., Turner D., Winder M. (2019). Ecological and functional consequences of coastal ocean acidification: Perspectives from the Baltic-Skagerrak System. Ambio.

[87] Kubota K., Yokoyama Y., Ishikawa T., Suzuki A., Ishii M. (2017). Rapid decline in pH of coral calcification fluid due to incorporation of anthropogenic CO_2_. Sci. Rep..

[88] Fujii M., Takao S., Yamaka T., Akamatsu T., Fujita Y., Wakita M., Yamamoto A., Ono T. (2021). Continuous Monitoring and Future Projection of Ocean Warming, Acidification, and Deoxygenation on the Subarctic Coast of Hokkaido, Japan. Front. Mar. Sci..

[89] Wanjeri V.W.O., Okuku E., Ngila J.C., Ndungu P.G. (2023). Effect of seawater acidification on physiological and energy metabolism responses of the common Cockle (Anadara antiquata) of Gazi Bay, Kenya. Mar. Pollut. Bull..

[90] Trull T.W., Passmore A., Davies D.M., Smit T., Berry K., Tilbrook B. (2018). Distribution of planktonic biogenic carbonate organisms in the Southern Ocean south of Australia: a baseline for ocean acidification impact assessment. Biogeosciences.

[91] Madkaiker K., Valsala V., Sreeush M.G., Mallissery A., Chakraborty K., Deshpande A. (2023). Understanding the seasonality, trends, and controlling factors of Indian Ocean acidification over distinctive bio-provinces. JGR. Biogeosciences.

[92] Sarma V.V.S.S., Paul Y.S., Vani D.G., Murty V.S.N. (2015). Impact of river discharge on the coastal water pH and pCO_2_ levels during the Indian Ocean Dipole (IOD) years in the western Bay of Bengal. Cont. Shelf Res..

[93] Shetye S.S., Naik H., Kurian S., Shenoy D., Kuniyil N., Fernandes M., Hussain A. (2020). pH variability off Goa (eastern Arabian Sea) and the response of sea urchin to ocean acidification scenarios. Mar. Ecol..

[94] Sharma D., Biswas H., Bandyopadhyay D. (2022). Simulated ocean acidification altered community composition and growth of a coastal phytoplankton assemblage (Southwest coast of India, eastern Arabian Sea). Environ. Sci. Pollut. Res. Int..

[95] Sharma D., Biswas H., Silori S., Bandyopadhyay D., Shaik A.u., Cardinal D., Mandeng-Yogo M., Ray D. (2020). Impacts of Zn and Cu enrichment under ocean acidification scenario on a phytoplankton community from tropical upwelling system. Mar. Environ. Res..

[96] Glibert P.M., Cai W.-J., Hall E.R., Li M., Main K.L., Rose K.A., Testa J.M., Vidyarathna N.K. (2022). Stressing over the complexities of multiple stressors in marine and estuarine systems. Ocean-Land-Atmosphere Res..

[97] Panchang R., Ambokar M. (2021). Ocean acidification in the Northern Indian Ocean: A review. J. Asian Earth Sci..

[98] Billé R., Kelly R., Biastoch A., Harrould-Kolieb E., Herr D., Joos F., Kroeker K., Laffoley D., Oschlies A., Gattuso J.-P. (2013). Taking action against ocean acidification: a review of management and policy options. Environ. Manage..

[99] Barton A., Hales B., Waldbusser G.G., Langdon C., Feely R.A. (2012). The Pacific oyster, Crassostrea gigas, shows negative correlation to naturally elevated carbon dioxide levels: Implications for near-term ocean acidification effects. Limnol. Oceanogr..

[100] Sheelagh McCarthy (2019).

[101] Hartmann J., West A.J., Renforth P., Köhler P., De La Rocha C.L., Wolf-Gladrow D.A., Dürr H.H., Scheffran J. (2013). Enhanced chemical weathering as a geoengineering strategy to reduce atmospheric carbon dioxide, supply nutrients, and mitigate ocean acidification. Rev. Geophys..

[102] Taylor L.L., Quirk J., Thorley R.M.S., Kharecha P.A., Hansen J., Ridgwell A., Lomas M.R., Banwart S.A., Beerling D.J. (2016). Enhanced weathering strategies for stabilizing climate and averting ocean acidification. Nat. Clim. Chang..

[103] Chopin T., MacDonald B., Robinson S., Cross S., Pearce C., Knowler D., Noce A., Reid G., Cooper A., Speare D. (2013). The Canadian Integrated Multi-Trophic Aquaculture Network (CIMTAN)—a network for a new ERA of ecosystem responsible aquaculture. Fisheries.

[104] Parker L.M., Ross P.M., O’Connor W.A. (2011). Populations of the Sydney rock oyster, Saccostrea glomerata, vary in response to ocean acidification. Mar. Biol..

[105] Albright R., Cooley S. (2019). A review of interventions proposed to abate impacts of ocean acidification on coral reefs. Reg. Stud. Mar. Sci..

[106] Howard J.L., Creed J.C., Aguiar M.V.P., Fourqurean J.W. (2018). CO_2_ released by carbonate sediment production in some coastal areas may offset the benefits of seagrass “Blue Carbon” storage. Limnol. Oceanogr..

[107] Cooley S.R., Ono C.R., Melcer S., Roberson J. (2016). Community-level actions that can address ocean acidification. Front. Mar. Sci..

[108] Green M.A., Waldbusser G.G., Reilly S.L., Emerson K., O’Donnell S. (2009). Death by dissolution: Sediment saturation state as a mortality factor for juvenile bivalves. Limnol. Oceanogr..

[109] Zheng H., Tan K., Zhang H., Ma H., Li S., Zheng H. (2022). Intraspecific hybridization as a mitigation strategy of ocean acidification in marine bivalve noble scallop Chlamys nobilis. Sci. Total Environ..

[110] Global Ocean Acidification Observing Network (2024). GOA-ON: The Global Ocean Acidification Observing Network. https://www.goa-on.org/.

