# Supplementary material for: Ocean acidification: Global perspectives and India’s path forward
Source: iScience. 2025 Oct 24;28(11):113852. doi: 10.1016/j.isci.2025.113852 (PMC12666105; doi:10.1016/j.isci.2025.113852)
Supplement: Data S1. Supplementary research data for the study [file mmc1.zip › Supplementary file/Table 1.docx]

**Table 1.** *Summary of key ocean acidification strategies categorized by type, mechanism of action, scalability, and global examples. This table provides a comparative overview of mitigation, adaptation, monitoring, and geoengineering approaches, highlighting their readiness levels and documented implementation sites to guide future research and policy actions.*

| **Strategy** | **Type** | **Mechanism of Impact on Ocean Acidification** | **Scalability / Technology Readiness** | **Location / Global Example** |
| --- | --- | --- | --- | --- |
| CO₂ Emission Reduction^98^ | Mitigation | Reduces atmospheric CO₂ concentrations, thereby slowing ocean acidification globally. Proven essential for long-term OA mitigation. | Global-scale policy integration; high readiness; critical for climate goals | UNFCCC, Kyoto Protocol, Paris Agreement |
| Monitoring & Early Warning Systems^21,99^ | Monitoring | Enable early detection and forecasting of acidification hotspots, supporting adaptive management and timely intervention. | Widely implemented; high readiness with ongoing improvements | Integrated Ocean Observing System (IOOS) |
| Coastal Pollution Control^100^ | Mitigation | Reduces eutrophication and hypoxia, mitigating local acidification caused by nutrient runoff. | Moderate scalability; requires regional coordination and infrastructure | Puget Sound, USA nutrient control strategies |
| Site-Specific Buffering^21,99^ | Adaptation | Stabilizes pH levels in aquaculture hatcheries using buffering agents, improving survival of early life stages. | Locally effective; widely used in aquaculture hatcheries | Whiskey Creek Shellfish Hatchery, USA |
| Marine Protected Areas (MPAs)^28^ | Adaptation | Reduces compounding stressors and protects biodiversity, enhancing ecological resilience to OA. | High policy acceptance; effective for biodiversity protection | Great Barrier Reef MPAs, California MPAs |
| Ocean Alkalinity Enhancement (OAE)^22,23^ | Geoengineering | Adds alkalinity to seawater, increasing buffering capacity and neutralizing excess acidity. | Emerging strategy; field trials ongoing; regulatory frameworks evolving | Experimental trials using lime (CaO) or olivine (Mg₂SiO₄), Mediterranean-based shipping alkalinization |
| Enhanced Chemical Weathering (EW)^101^ | Geoengineering | Accelerates natural weathering to enhance alkalinity in riverine runoff, indirectly mitigating OA. | Pilot-stage; land-sea coupling requires further research | Spreading ground silicate rocks on land |
| Enhanced Terrestrial Weathering^102^ | Geoengineering | Increases carbon sequestration and ocean buffering over longer timeframes through silicate application. | Under exploration; geochemically promising; low readiness | Application of silicate rocks (e.g., dunite, basalt) over tropical regions |
| Integrated Multi-Trophic Aquaculture (IMTA)^103^ | Adaptation | Improves carbon uptake and nutrient cycling, locally buffering seawater pH. | Proven at small scale; expanding in Asia and Europe | Canada, Norway (fish, seaweed, shellfish integration) |
| Selective Breeding for OA Resilience^104,105^ | Adaptation | Develops aquaculture species with greater physiological tolerance to acidified conditions. | Active in aquaculture R&D; high species-specific potential | Sydney rock oysters bred for pH tolerance |
| Seagrass & Algae Restoration^105,106^ | Adaptation | Increases local pH via photosynthesis and provides critical habitat for calcifying species. | Ecosystem-based; scalable with conservation support | Seagrass meadows near coral reefs, Florida Reef Tract |
| Ecosystem-Based Fisheries Management^107^ | Adaptation | Maintains trophic balance and ecosystem health, reducing stress that worsens OA impacts. | Policy-backed in many regions; supports OA resilience indirectly | US West Coast Fisheries Management |
| Local Habitat Restoration with Shell Reefs^108^ | Adaptation | Reintroduces shell material that dissolves and buffers pH, enhancing conditions for calcifiers. | Community-scale success; integrates with coastal conservation | Chesapeake Bay oyster reef restoration |
| Artificial Reef Structures^105^ | Adaptation | Provides carbonate substrate and refuge, which may indirectly aid calcifiers in acidified areas. | Locally implemented; dual benefits for tourism and habitats | Reef Balls, Electrochemical deposition reefs |
| Hybridization for OA Resilience^109^ | Adaptation | Produces offspring with improved survival under low pH, tested in lab and aquaculture trials. | Lab-tested; early stage in field application | Intraspecific hybrids of *Chlamys nobilis* (China) |
